# Identifying intact *N*-glycopeptides from tandem mass spectrometry data using StrucGP

**DOI:** 10.52601/bpr.2022.220010

**Published:** 2022-12-31

**Authors:** Jiechen Shen, Zexuan Chen, Shisheng Sun

**Affiliations:** 1 College of Life Sciences, Northwest University, Xi’an 710069, China

**Keywords:** Protein glycosylation, Glycoproteomics, Mass spectrometry, StrucGP, Glycan structure

## Abstract

Protein glycosylation is of great importance in many biological processes. Glycosylation has been increasingly analyzed at the intact glycopeptide level using mass spectrometry to study site-specific glycosylation changes under different physiological and pathological conditions. StrucGP is a glycan database-independent search engine for the structural interpretation of *N*-glycoproteins at the site-specific level. To ensure the accuracy of results, two collision energies are implemented in instrument settings for each precursor to separate fragments of peptides and glycans. In addition, the false discovery rates (FDR) of peptides and glycans as well as probabilities of detailed structures are estimated. In this protocol, the use of StrucGP is demonstrated, including environment configuration, data preprocessing as well as result inspection and visualization using our in-house software “GlycoVisualTool”. The described workflow should be able to be performed by anyone with basic proteomic knowledge.

## INTRODUCTION

### Importance of glycoproteomics

Glycosylation is one of the most important post-translational modifications of proteins and plays crucial roles in protein localization, stability, enzymatic activities, and protein–protein interactions (Spiro 2002). The occurrence and development of many diseases are often accompanied by changes in glycosylation, thus searching these abnormal glycopeptides against overall glycosylation is critical for finding new biomarkers and therapeutic targets (Dube and Bertozzi 2005; Kellokumpu* et al.* 2002). For decades, high-sensitivity mass spectrometry-based glycoproteomics has grown to be a powerful approach for protein glycosylation analysis. The information of peptide sequence, glycosylation site and corresponding glycan structure from one single glycopeptide could be acquired simultaneously. Both quantity and quality of glycopeptide identification continue to increase with the optimizations of the workflow, especially with the explosive development of computational search algorithms for interpreting glycopeptides within the last few years (Fang* et al.* 2022; Lu* et al.* 2020; Lynn* et al.* 2015; Polasky* et al.* 2020; Shen* et al.* 2021; Xiao and Tian 2019; Zeng* et al.* 2021). It is foreseeable that in the near future, glycobiology will be taken to a whole new level with the large-scale functional analysis of site-specific glycosylation.

### Importance of structural interpretation of glycans on glycopeptides

Specific glycan structures play a crucial role in the functioning of proteins to which they are attached. The human ABO blood group, which is determined by the presence or absence of A and B trisaccharide antigens (Yamamoto* et al.* 1990), might be one of the best-known examples. Another example of glycan epitope is Lewis^x^, which can be attached to the core structures of *N*- and *O*-glycans, and serves as a prognostic marker for several malignant tumors such as the bladder (Konety* et al.* 1997), lung (Kadota* et al.* 1999) and medulloblastoma cancers (Read* et al.* 2009). Core fucosylation, where a fucose bears in the innermost GlcNAc of *N*-glycans, was extensively reported to be a potential prognostic biomarker for many cancers (Bastian* et al.* 2021).

### The advantages of StrucGP

StrucGP has two major advantages: interpreting site-specific *N*-glycans at a structural level and analyzing new glycan structures thanks to the implemented glycan database-independent strategy.

To achieve better structural interpretation of *N*-glycans on glycopeptides, it is highly recommended that each glycopeptide precursor is fragmented by two individual HCD energies (*e*.*g*., 20% HCD and 33% HCD) in mass spectrometry analysis. Under the low HCD energy, the glycan portion of the glycopeptide can be fragmented into B/Y ions, which is essential for glycan structure determination. While under the high HCD energy, the peptide portion of the glycopeptide would be fragmented into b/y ions for peptide sequence identification. In addition, StrucGP can also analyze the mass spectrometry data generated with stepped collisional energies (SCE), especially for the samples where branching structures have been established.

In terms of identification strategy, StrucGP adopts a modular strategy, by which an *N*-glycan is first separated into the core structure, glycan type, as well as branch structures, and each module is determined based on the related characteristic B/Y ions. Benefiting from the modularized strategy, StrucGP can identify glycan structure and composition without building a sample-specific glycan database, and thus can identify new glycans that do not exist in the regular glycan database. To control the identification accuracy, in addition to the regular FDR estimation for peptides, FDR is also estimated at the glycan level by adding random masses (or a random mass) to precursors to generate “decoy spectra”. Moreover, the probability of each detailed sub-structures is also calculated to evaluate the quality of detailed structures identified from each spectrum. Also, identified glycopeptide spectra can be visualized using the GlycoVisualTool software.

### The focus of this Protocol

This protocol aims to guide researchers to perform glycoproteomics data analysis using StrucGP, including preparation of the search environments, downloading protein databases, searching MS/MS files, and inspecting and visualizing the results. The operation of each part is described with as many details as possible, and in some critical steps, we enumerate several situations that may affect data quality, and provide possible solutions. Although this protocol is mainly focused on the identification of intact glycopeptides (IGP), quantitation analysis of IGP with TMT or iTRAQ labelling is also allowed by setting the label type in the corresponding step. Raw files used in this protocol are available at the ProteomeXchange Consortium (http://proteomecentral.proteomexchange.org) with access code PXD025859.

## SETUP

### MS data requirement

For each precursor, HCD-MS/MS with two different energies (a lower energy for the glycan portion fragment and a higher energy for the peptide backbone fragment), or stepped collision energies are required. Other MS parameters used for the LC-MS/MS analysis are recommended in Table 1.

**Table 1:** MS parameters used for the LC-MS/MS analysis

Setting	Value	Notice
Full MS		
Resolution	120,000	
Scan range	375–2000 *m*/*z*	
RF lens	40%	A higher RF level increases the transmission of high *m*/*z* ions. For glycopeptides, *m*/*z* is generally higher than peptides.
Filters		
Charge states	2–7	
Dynamic exclusion	20 s	
Mass tolerance	10 ppm	
Mass range	700–2000 *m*/*z*	Glycopeptides’ mass range is generally between 1500–4500 Da, with precursor charge states from two to five. This *m*/*z* range can avoid many peptide peaks.
High-energy dd-MS^2^		
Collision energy	33%	
Isolation window	2 *m*/*z*	
Resolution	30,000	
Scan range	120–3000 *m*/*z*	This setting can include the oxonium ions at138.055 *m*/*z*, and many Y ions higher than 2000 *m*/*z*.
Maximum injection time	100 ms	More ions for better fragmentation.
AGC target	200,000	More ions for better fragmentation.
Low-energy dd-MS^2^		
Collision energy	20%	
Isolation window	0.7 or 2 *m*/*z*	A narrow isolation window (0.7 *m*/*z*) avoids the interference of co-eluted precursor ions for the determination of glycan structure (higher spectral quality) while a wider window (2 *m*/*z*) identifies more glycopeptides.
Resolution	30,000	
Scan range	120–3000 *m*/*z*	
Maximum injection time	100 ms	
AGC target	200,000	
RF: radio frequency; AGC: automatic gain control. We take an Orbitrap Fusion Lumos instrument as an example here		

### Hardware and software requirements

• Windows 10 or Windows 7 Service Pack 1 above, Windows Server 2012 or above

• Intel or AMD x86-64 processor, 4.6 GHz processor with 16 GB RAM

• ProteoWizard at version 3 (including the MSConvert module) is available at ProteoWizard: Home (sourceforge.io)

• .NET Framework 4.7.2 or a higher version is available at https://dotnet.microsoft.com/download/thank-you/net472

• StrucGP (version 1.1.1) , as well as GlycoVisualTool (version 1.0.0), can be freely downloaded from https://zenodo.org/record/6543379#.YunkdWNByUk and https://github.com/Sun-GlycoLab/StrucGP/releases

### Installation

#### StrucGP

StrucGP can be used without installation.

#### GlycoVisualTool

**Figure 1:**
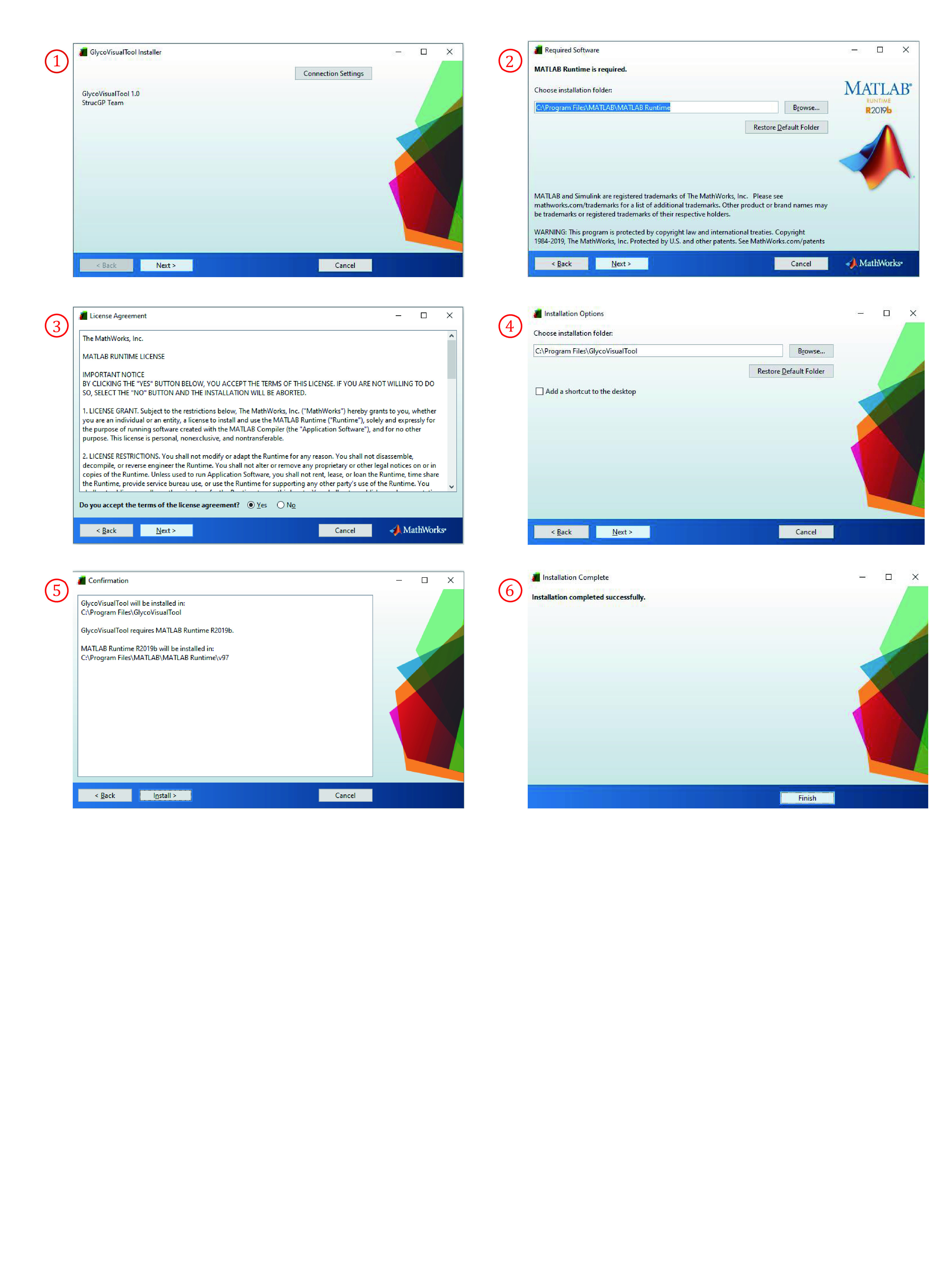
The installation procedure of GlycoVisualTool

(1) Double click MyAppInstaller_mac.exe in a folder named “for_redistribution” to start installing GlycoVisualTool.

(2) Click “Next” shown in Fig.1 after you accept or change these following settings.

(3) GlycoVisualTool is developed by MATLAB and relies on Runtime. When the up-to-date Runtime is needed, a download link will show up as you install GlycoVisualTool.

### Preparation before starting StrucGP

#### Step 1: Obtain a license for StrucGP

1.1 Double click main.exe in the StrucGP folder.

**Figure 2:**
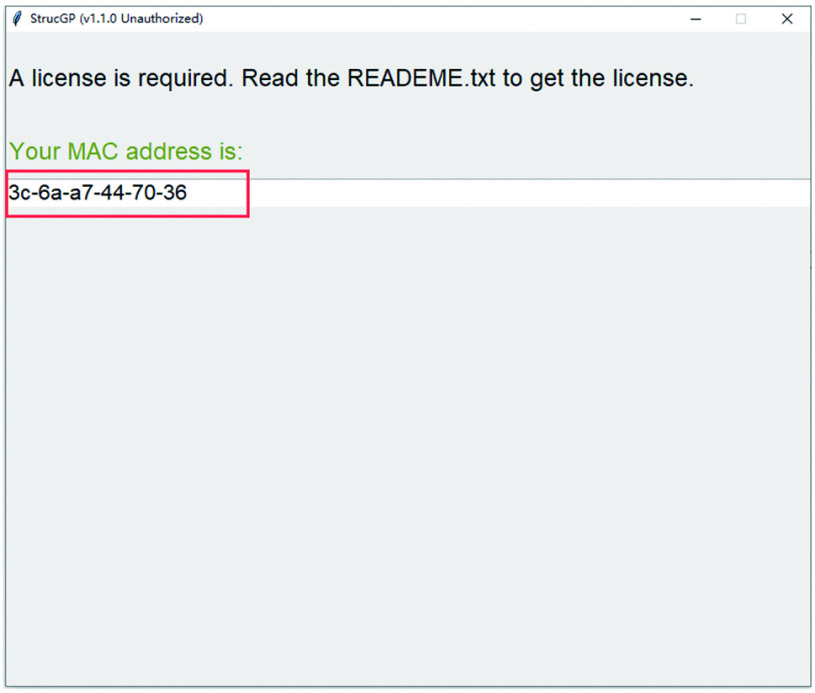
Getting MAC address

1.2 Copy the “MAC address” from the interface (Fig. 2) and then send an email to sun_glycolab@126.com to get a license file named “StrucGP_license.lic”.

1.3 Put the license file into the same directory as main.exe.

**Figure 3:**
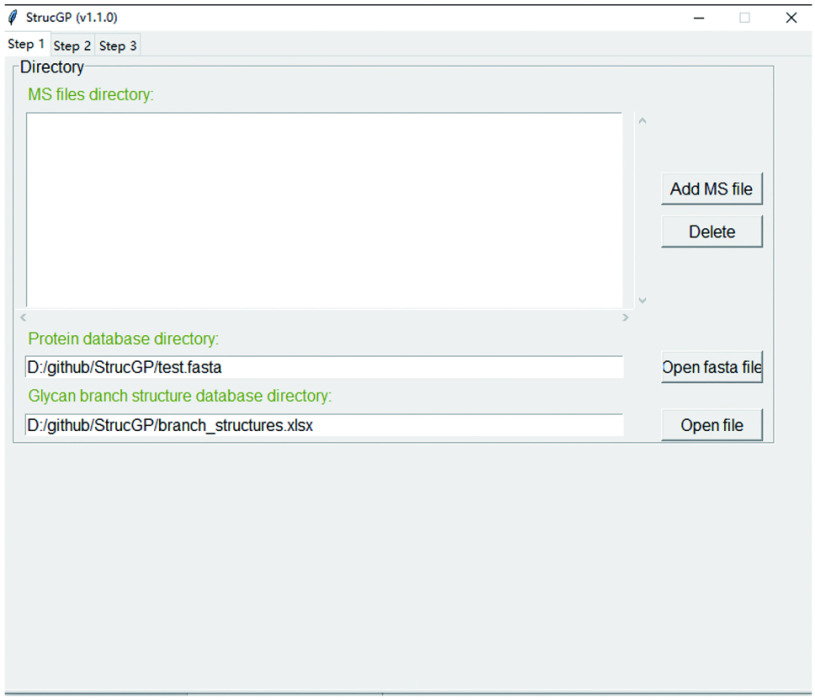
The interface after opening StrucGP correctly

1.4 Double click main.exe again, and an interface as Fig. 3 will show up once StrucGP works correctly.

**[NOTE]** To avoid potential errors, only use English characters and underscores in the directory of StrucGP, all file names, and file paths when running StrucGP.

#### Step 2: Converting MS data from “RAW” into “mzML” format

**Figure 4:**
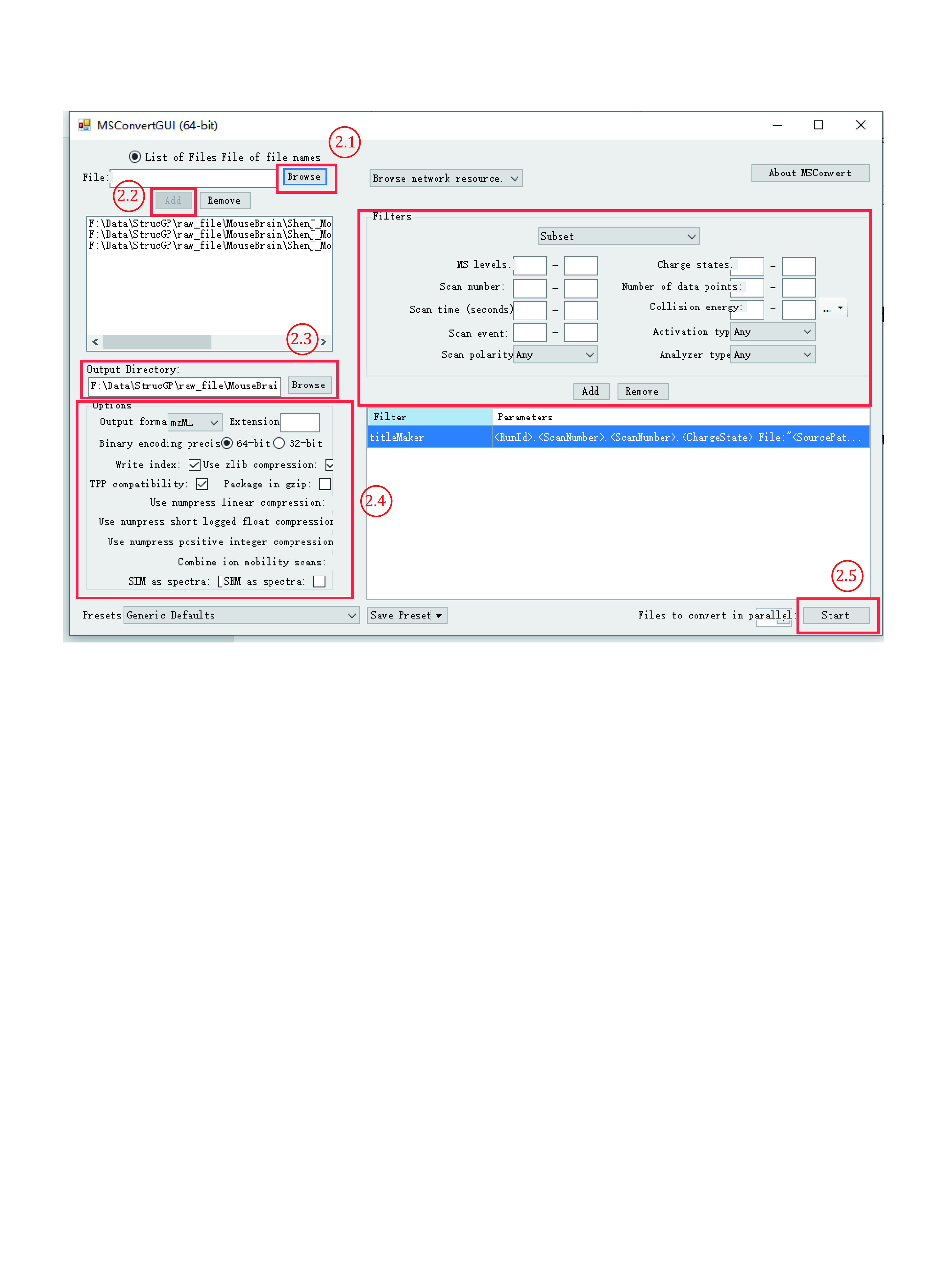
Format conversion using MSConvert

2.1 Open the MSConvert tool of ProteoWizard (Fig. 4).

2.2 Click “Browse” in the upper left to choose desired .raw file(s).

2.3 If only one .raw file is selected, click “Add” to add the .raw file to the candidate convert list box under the “Add” button.

2.4 Set “Options” and “Filters” as an example (or as defaults).

2.5 Click “Start” to start the conversion.

#### Step 3: Download protein databases from UniProt (Optional)

To perform searches, a protein database in “fasta” format is required. We highly recommend users download protein or proteome databases via UniProt. It is also feasible to DIY a protein database if needed.

**Figure 5:**
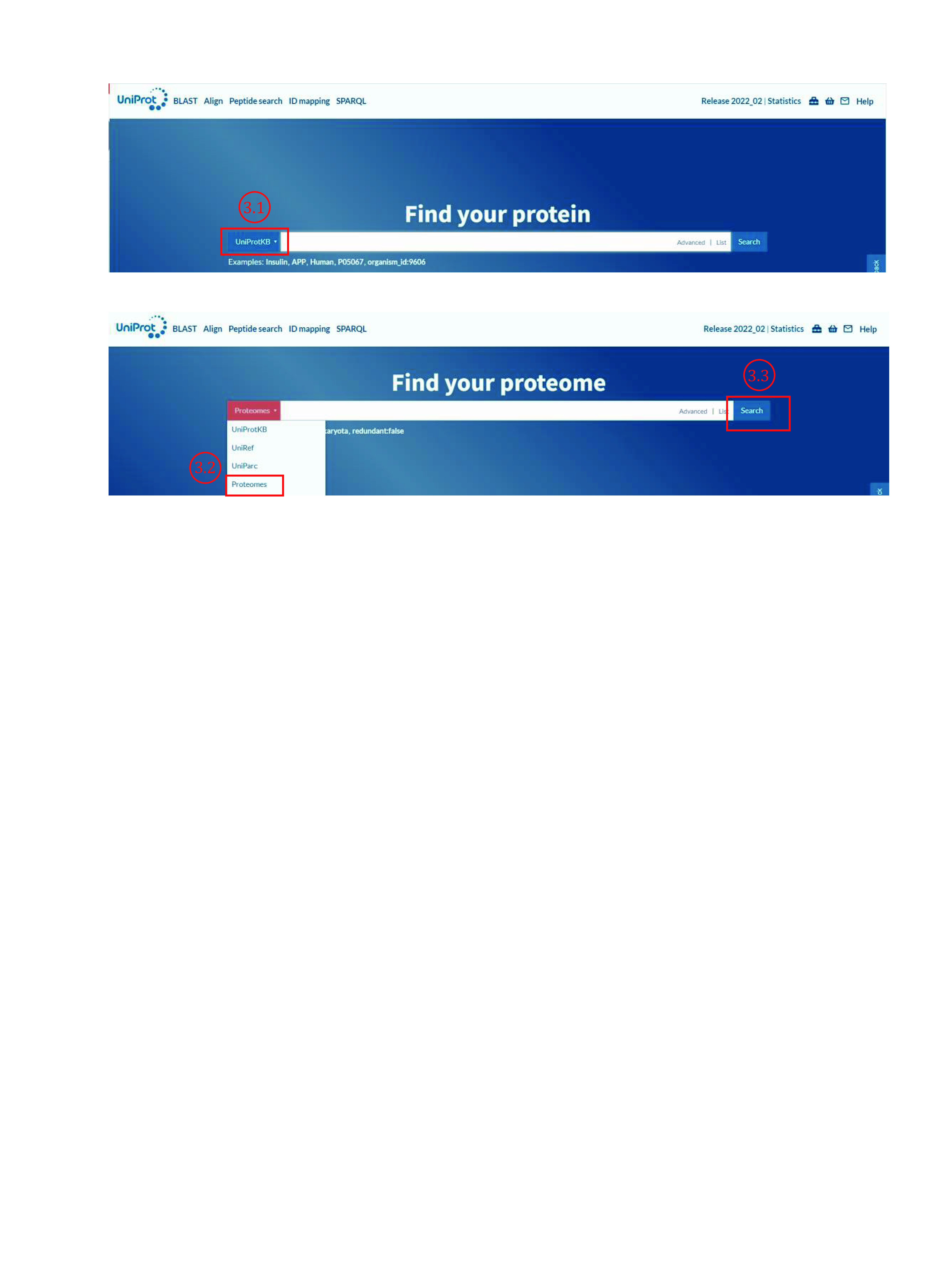
Download protein database from UniProt (1)

3.1 Open the website of UniProt (https://www.uniprot.org/) (Fig. 5).

3.2 Click “Proteomes” in the pop-up list.

3.3 Type species name (*e*.*g*., *mus musculus*) and then click “Search”.

3.4 Click the proteome ID of the desired organism (*e*.*g*., UP000000589 for mouse database).

3.5 Click the number (*e.g*., 55353) in the back of “Protein count”.

**Figure 6:**
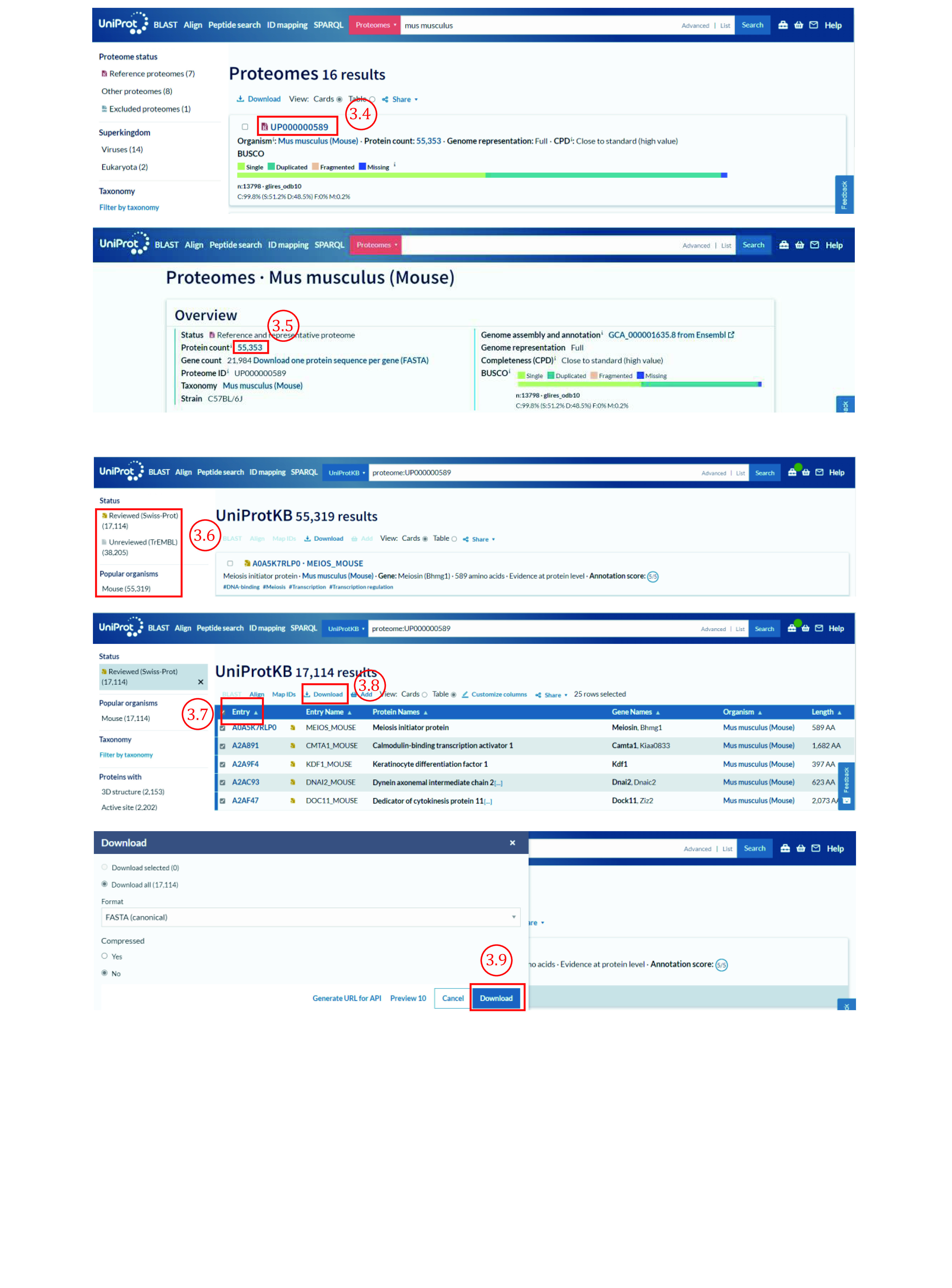
Download protein databases from the UniProt (2)

3.6 Choose the annotation type of protein (reviewed, unreviewed, or both two types) on the newly opened page (Fig. 6).

**[NOTE]** It is recommended to use the reviewed database. Firstly, proteins in the reviewed database are generally of high confidence. In addition, according to our experience, a larger protein database in one search will lead to a smaller number of PSMs by a cutoff of 1% peptide FDR.

3.7 Tick the box to the left of “Entry” to choose all proteins.

3.8 Click “Download”.

3.9 Choose “Download all” with the format “FASTA (canonical)”, then click “Go” to start a download.

### Setting parameters for a search (time cost: minutes)

#### Step 4: Open the StrucGP software

**Figure 7:**
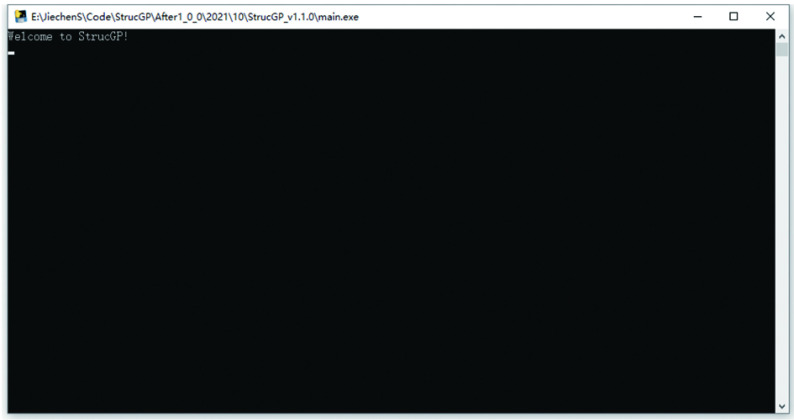
Console window of StrucGP

If you have already done all preparations documented above, then double click “main.exe” to start StrucGP. Besides the main interface of StrucGP (Fig. 3), you will also see a console window like Fig. 7, where the running status of the software and possible error messages will be displayed.

#### Step 5: Select MS/MS file and database for searching

5.1 Click “Add MS file” to add desired .mzML files. The number of added MS files in one search is not limited.

**[NOTE]** Search results will be generated in the same path as the .mzML file. Before starting a new search, make sure that the old search results will not be overwritten.

5.2 Click “Open .fasta file” to select a protein database for this search.

**[CRITICAL STEP]** The decoy peptides are generated from peptides in the peptide database with a proportion of 1:1. Too few peptides in the database will make the FDR of the peptides unreliable. For a more accurate result, always put the target protein sequences into a large database (more than 100 proteins) when searching for a single protein MS data.

**Figure 8:**
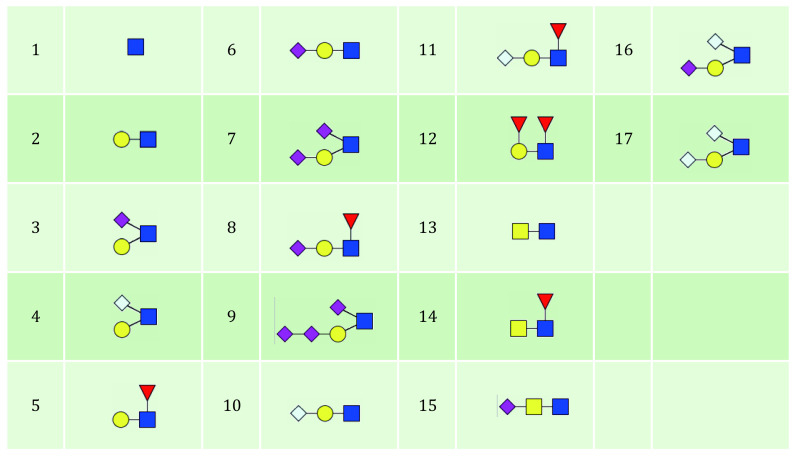
Branch structures in current version of StrucGP

5.3 Click “Open file” in the lower right corner to choose the glycan branch structure database. The branch structure database can be freely downloaded from the same site as StrucGP which is named “branch_structures.xlsx”. There are a total of 17 branch structures in the default branch structure database (Fig. 8), all of which were verified manually in mouse and human MS/MS data. Only branch structures in the database can be identified. To add new structures, *e*.*g*., branch structure 14 in Fig. 8.

**Figure 9:**
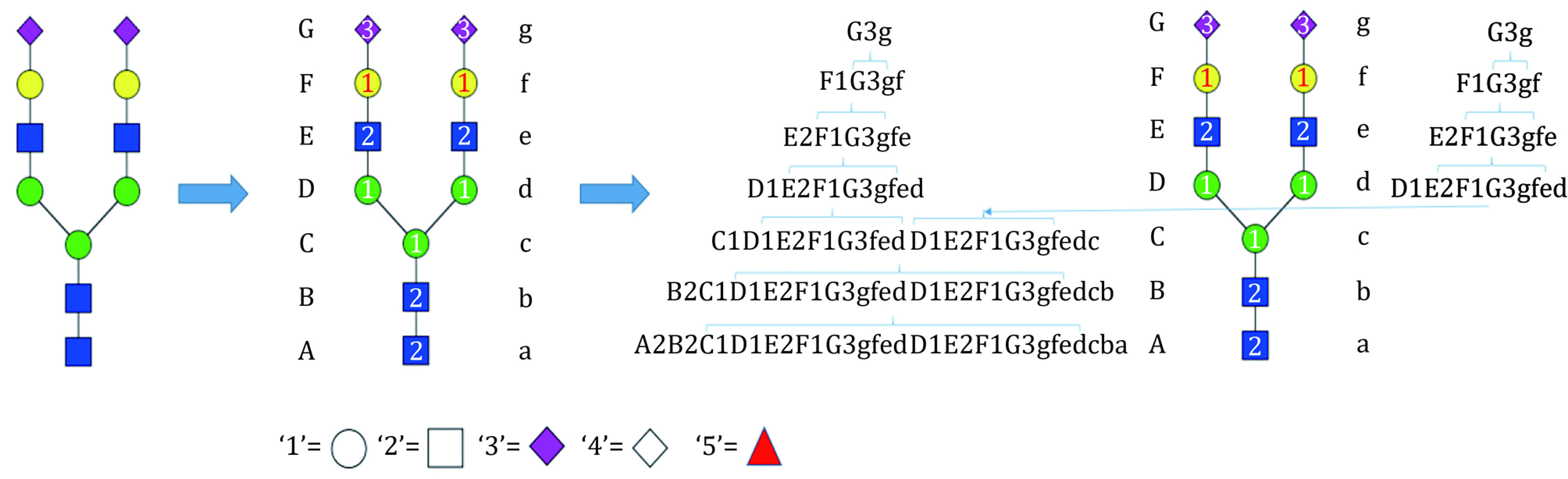
The glycan structure code of StrucGP. Uppercase and lowercase letters represent the beginning and end of a layer, respectively. "A" represents the first layer of the glycan from the reducing end, "B" represents the second layer of the glycan from the reducing end, *etc*. Numbers "1" to "5" represent Hex, HexNAc, NeuAc, NeuGc, and dHex, respectively

(1) Numbering new structures following the existing ones, *e*.*g*. 18.

(2) Structure coding: according to the encoding of the glycan structures (Fig. 9). In this example the structured coding is “E2F2fF5fe”.

(3) Branch structure: add the composition of this structure. For example, the composition for this example should be N2F1, which stands for glycans with two HexNAc and one Hex. Normally, Hex, HexNAc, NeuAc, NeuGc, and dHex are abbreviated as H, N, S, G, and F, respectively.

(4) Essential ions (optional): the ions must appear in the spectra of this structure. If any ion in “essential ions” is missing from one spectrum, then this structure will be filtered out in the glycan structure search for this spectrum. The format of these ions is the same as in “Branch structure”.

(5) Ions can’t appear (optional): ions should not appear in the spectra for this structure. The branch structure would be filtered out if any ions in this list are detected in the spectrum.

(6) Not essential ions (optional): these ions are used only for visualization.

**[NOTE]** When searching for data from different samples, there is no need to delete branch structures that theoretically do not exist in the samples.

#### Step 6: Configure experiment settings

**Figure 10:**
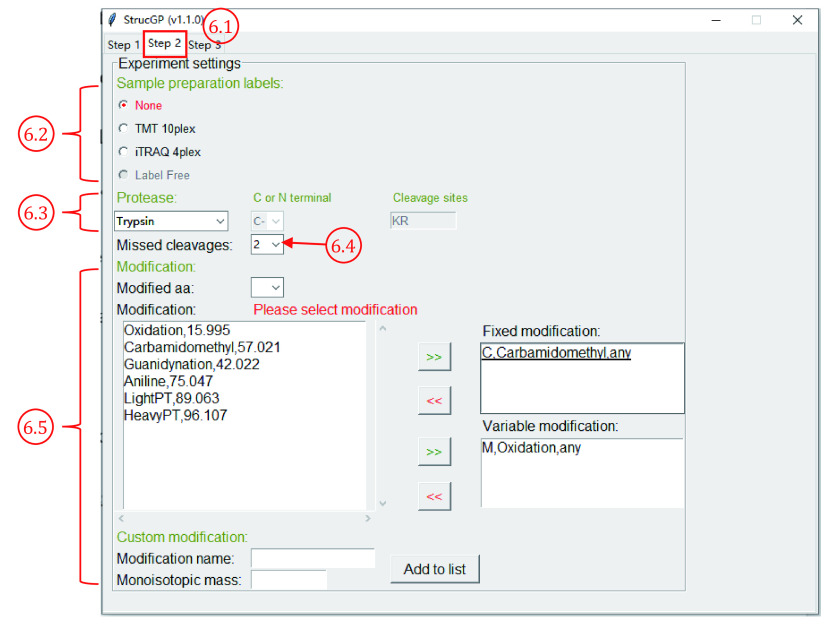
Configure experiment settings

6.1 Click “Step 2” to switch to the “Experiment settings” table (Fig. 10).

6.2 Select label type “None”, “TMT 10plex” or “iTRAQ 4plex”. The intensities of label ions will be included in the result file if “TMT 10plex” or “iTRAQ 4plex” is selected for quantification.

**[CRITICAL STEP]** For glycopeptide identifications using StrucGP, it is highly recommended to set low and high collision energy (*e*.*g*., 20% and 33% collision energy) for fragmentations of each precursor during mass spectrometry analysis to interpret glycan structure and peptide sequence separately. If you are using TMT 10plex for quantification, it’s recommended to set higher collision energy for peptide search (*e*.*g*., 37% collision energy) but keep the low collision energy unchanged (*e*.*g*., still keeping 20% collision energy for glycan structure interpretation).

6.3 Click the drop-down list under “Trypsin” to select protease, if your protease is not in the list, choose “Other protease”, and specify cleavage sites and the cutting directions (C-terminal or N-terminal).

6.4 Click the drop-down list right to “Missed cleavages”. The number of missed cleavages means the maximum number of missed sites in a peptide that will be generated *in silico* from the selected protein database. The size of the peptide database will increase dramatically as the number of misses increases; thus, it is recommended to set missed cleavages of no more than two.

6.5 Check out the modification setting of peptides. Text boxes under “Fixed modification” and “Variable modification” indicate the currently set fixed and variable modifications, respectively. Fixed modification of carbamidomethyl on cysteine (C, +57 Da) and variable modification of oxidation on methionine (M, +16 Da) are set as defaults. To add modifications for peptide search, click the drop-down list right to “Modified aa” to select the amino acids to be modified and click “**>>**” to add fixed or variable modification. If the modification of interest is not in the modification list, customized modifications can be added with a specific modification name and monoisotopic mass, then click “Add to list” to add a modification to the modification list.

#### Step 7: Configure search settings

**Figure 11:**
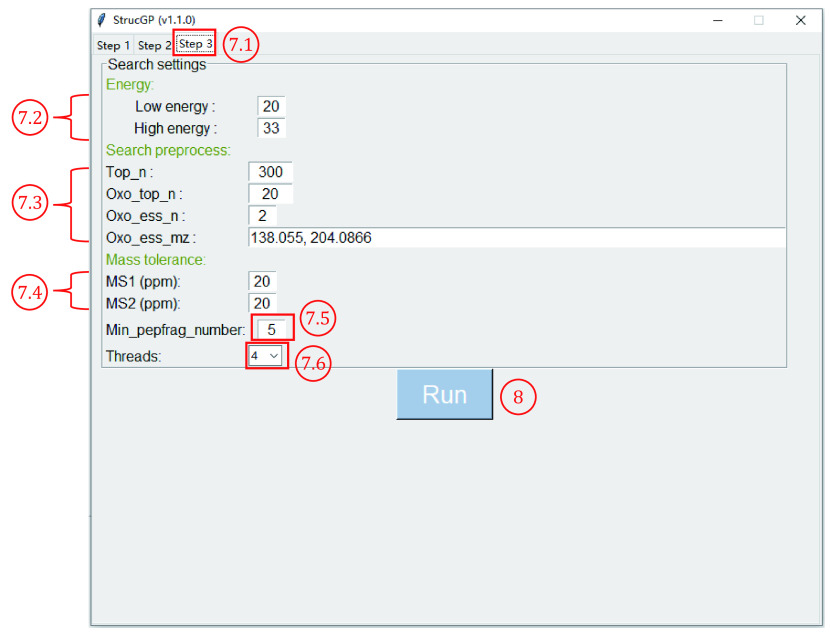
Configure search settings

7.1 Click “Step 3” to switch to the “Search settings” table (Fig. 11).

7.2 Specify collision energy for interpreting glycopeptides. Low- and high-energy spectra will be used to identify glycan structure and peptide sequence, respectively. If stepped collision energy (SCE) was used, *e*.*g*., 20-30-40 SCE, both the high and low energy should be set as 30. If the middle energy is not an integer, *e*.*g*., 20-26.5-33, the high and low energy should be set as its integer part (*i*.*e*., 26).

**[CRITICAL STEP]** The low and high energy specified here need to be set in equipment settings when performing mass spectrometry analysis.

7.3 Specify parameters for screening glycopeptide spectra. The meaning of each parameter is listed as follows:

(1) “Top_n”: The number of top peaks in high energy spectra that will be selected for peptide sequence searching (the default setting is 300, usually 200–500). Relatively small ‘Top_n’ can effectively help avoid random matching from noise peaks.

(2) “Oxo_top_n”, “Oxo_ess_n”, “Oxo_ess_mz”: parameters that filter N-glycopeptide spectra by specified oxonium ions. “Oxo_top_n” indicates that only the top n peaks will count as oxonium ions (many low abundant peaks may also occasionally match these oxonium ions, but these low abundant ions will not be considered as oxonium ions). “Oxo_ess_mz” and “Oxo_ess_n” indicate the oxonium ions for screening and the minimum required number of oxonium ions. These three parameters are set as follows by default: “Oxo_top_n” as “20”, “Oxo_ess_n” as “2”, and“Oxo_ess_mz” as “138.055, 204.087”, meaning that in the high energy spectrum of one candidate spectra pair, the top 20 peaks must contain at least two peaks within “Oxo_ess_mz” (in this case, *i*.*e*., both 138.055 and 204.087 must exist), otherwise, this spectra pair would be filtered out.

**[CRITICAL STEP]** Make sure that “Oxo_ess_mz” are within the *m/z* range of spectra.

7.4 Set mass tolerances for matching peaks in MS1 and MS2 spectra. We recommend a 20 ppm tolerance for both types of spectra.

**[CRITICAL STEP]** Make sure mass tolerance for MS2 is no more than 40 when using TMT 10*plex*. Because the difference between the nearest two labels is approximately 50 ppm.

7.5 “Min_pepfrag_number”: Minimum number of peptide fragments, one peptide will be filtered out if the number of peptide fragments in a spectrum is less than “Min_pepfrag_number”.

7.6 Click the drop-down list right to “Threads” to select the number of parallel processes for this search. The maximum number of parallel processes corresponds to the number of cores in your CPU.

**[NOTE]** To make a quicker search, set this number to no more than RAM divided by eight, *e*.*g*., if your RAM is 32 GB, it is best not to set this value greater than four.

#### Step 8: Start searching

**Figure 12:**
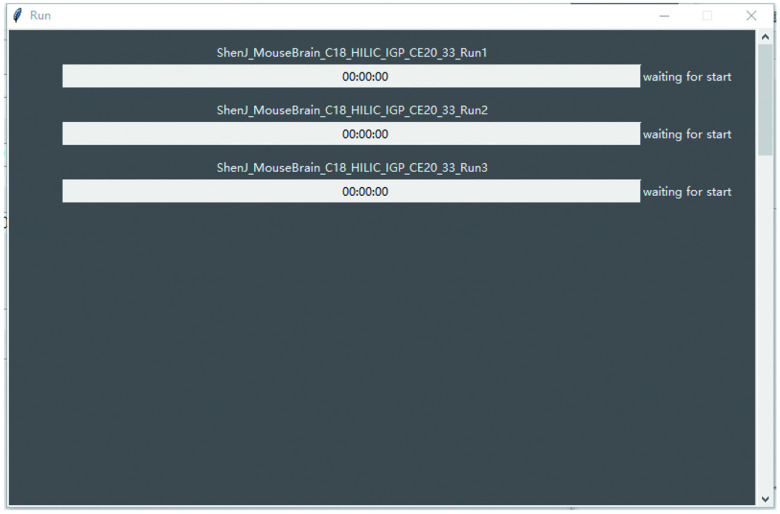
Window showing search progress

Click “Run” to start searching. If StrucGP works correctly, a new window will pop up showing the process within a few seconds (Fig. 12). The search will take a few minutes to hours, depending mainly on the number of glycopeptide spectra and MS files to search. The console window of StrucGP will show a detailed message when a program error occurs.

**[? TROUBLE SHOOTING]**

### Result check (time cost: minutes)

After the search is finished, there will be three result files in the same directory for each MS file, they are “MSFileName_result.xlsx”, “MSFileName_result_AllSpectra.xlsx” and “MSFileName.csv”.

**Figure 13:**
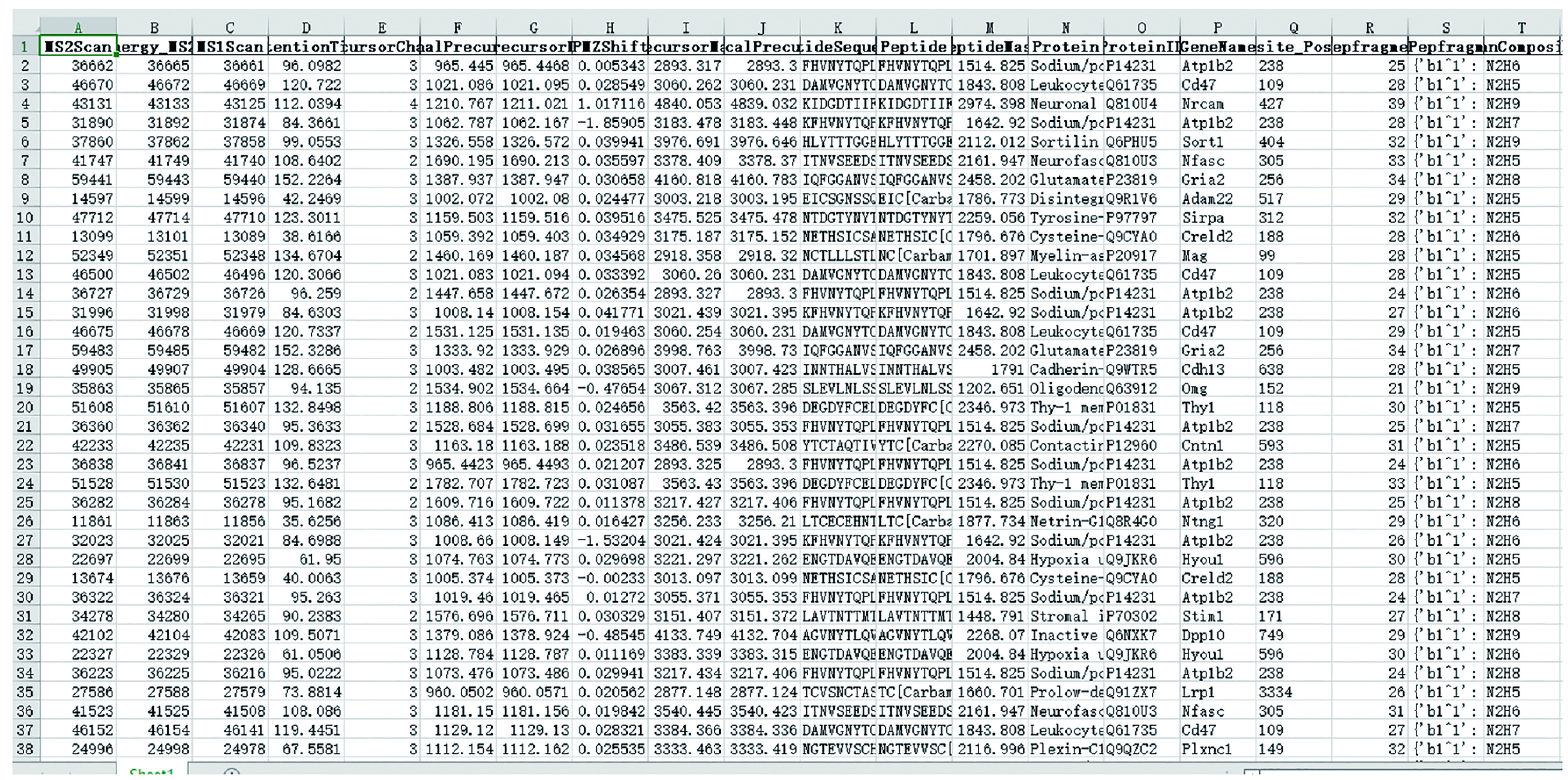
Screenshot of “MSFileName_result.xlsx”

**Figure 14:**
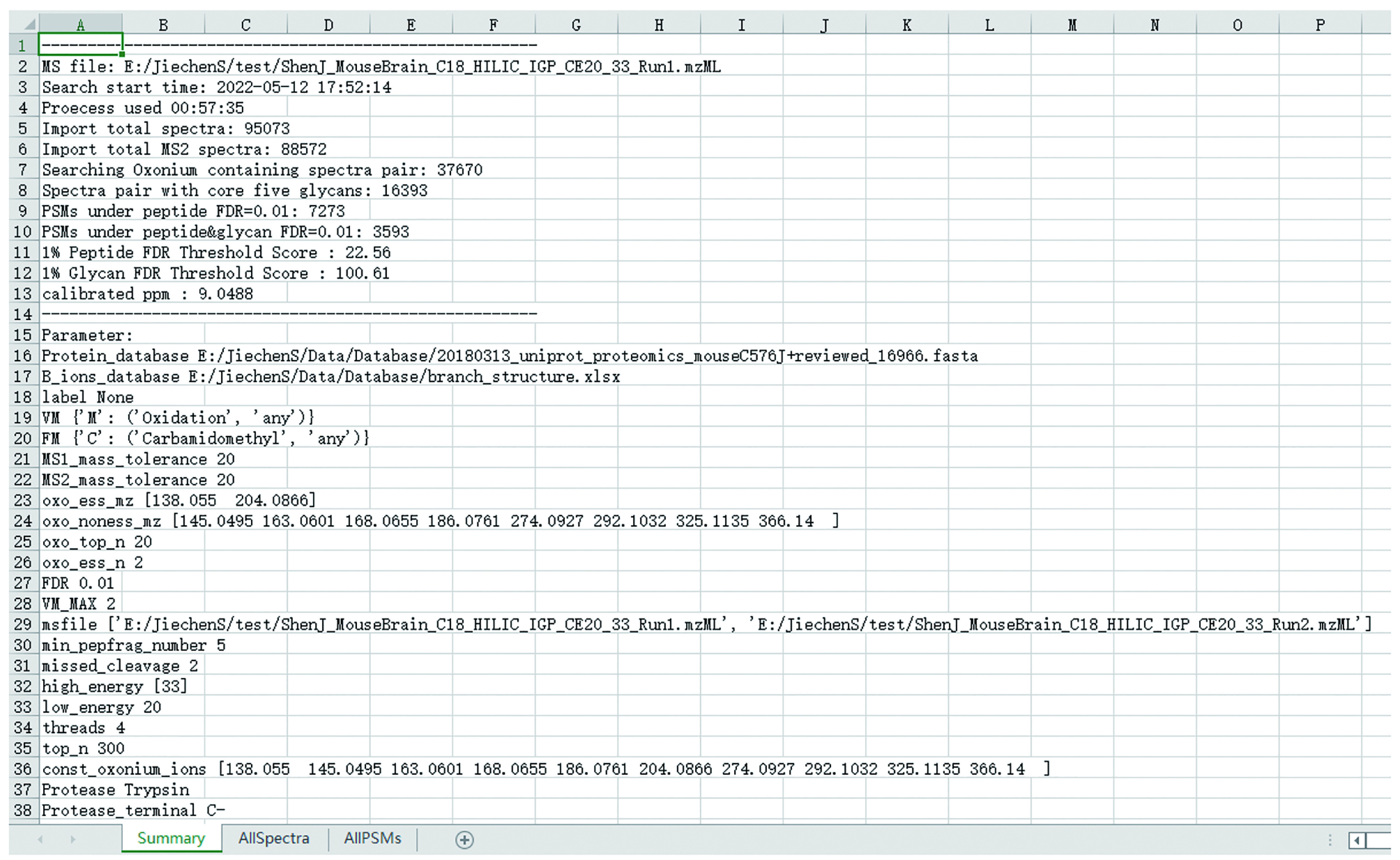
Screenshot of “MSFileName_result_AllSpectra.xlsx”

(1) “MSFileName_result.xlsx”: Result table with identified glycopeptides within 1% FDR at both peptide and glycan levels (Fig. 13).

(2) “MSFileName_result_AllSpectra.xlsx”: Include operating parameters (sheet ‘Summary’), death note of spectra pairs (The reason why the spectrum is filtered, sheet ‘AllSpectra’), and matched PSMs before screening FDR (sheet ‘AllPSMs’) (Fig. 14);

(3) “MSFileName.csv”: For visualizing and annotating all spectra pairs in “MSFileName_result.xlsx” by using GlycoVisualTool introduced below.

#### Step 9: Check searching profiles (Optional)

Double click “MSFileName_result_AllSpectra.xlsx”. There are three worksheets in this file, *i*.*e*., “Summary”: including an overview of this MS file and parameters used in this search, “AllSpectra’”: death note of spectra pairs (The reason why the spectrum is filtered), “AllPSMs”: all PSMs of glycopeptide with at least the peptide sequence matched (without peptide FDR filtering).

9.1 Worksheet “Summary” (Fig. 14)

(1) “MS file”. Detailed directory of the MS file.

(2) “Search start time” and “Process used time”. The exact time StrucGP started searching this MS file and its running time.

(3) “Import total spectra”. The number of spectra imported for search.

(4) “Import total MS2 spectra”. The number of MS2 spectra imported for search.

(5) “Searching Oxonium containing spectra pair”. The number of spectra pairs after oxonium ions screening (Qualified conditions are set in Step 7.3).

(6) “Spectra pair with core five glycans”. The number of spectra pairs that could match at least one Y1 candidate in low energy spectra. The number should be about half of the number of spectra pairs after oxonium ions screening, and if the number is too low, it’s better to readjust your mass spectrometry settings to improve the quality of low-energy spectra.

(7) “PSMs under peptide FDR0.01” and “PSMs under peptide&glycan FDR = 0.01”. The number of spectra pairs remaining after filtering with 1% FDR cutoff for peptides and/or glycans. The proportions of spectra pairs filtered in these two steps may vary from sample to sample, *e*.*g*., samples with a high proportion of high mannose glycans will filter fewer spectra than samples with a low proportion of high mannose glycans. While for the screening of peptide FDR, it may depend more on the quality of high-energy spectra, which are used for matching peptide sequences against the database.

(8) “1% Peptide FDR Threshold Score” and “1% Glycan FDR Threshold Score”. The scores of peptides and glycans at the threshold of 1% peptide and glycan FDR, respectively.

**[CRITICAL STEP]** Due to the glycan database independent approach of StrucGP, the accuracy of FDR estimation of glycans is based on the number of identified spectra. The more spectra are identified, the more accurate the glycan FDR estimation is. Therefore, it is highly recommended to set an additional glycan score threshold (*e*.*g*., 50) to exclude glycans that are obviously wrong.

(9) “calibrated ppm”. The average mass error of precursors of top 10% PSMs. If it is more than 10, calibrate the instrument or set a higher tolerance for searching.

9.2 worksheet “AllSpectra”. This sheet is generated to help those who would like to optimize mass spectrometry settings or sample handling strategies. Column “ScanNumber” means scan numbers of high energy spectra (set in 7.2) of spectra pairs. Column “DeathNote” means the filter reasons for corresponding spectra pairs, and the “pass” in “DeathNote” means the spectra passed all screenings before FDR estimation.

9.3 worksheet “AllPSMs”. All identified spectra PSMs before screening FDR. For those who would like to manually inspect spectra of interest that not matched any glycan or could not pass peptide or/and glycan FDR estimation.

#### Step 10: Check the filtered result

**Figure 15:**
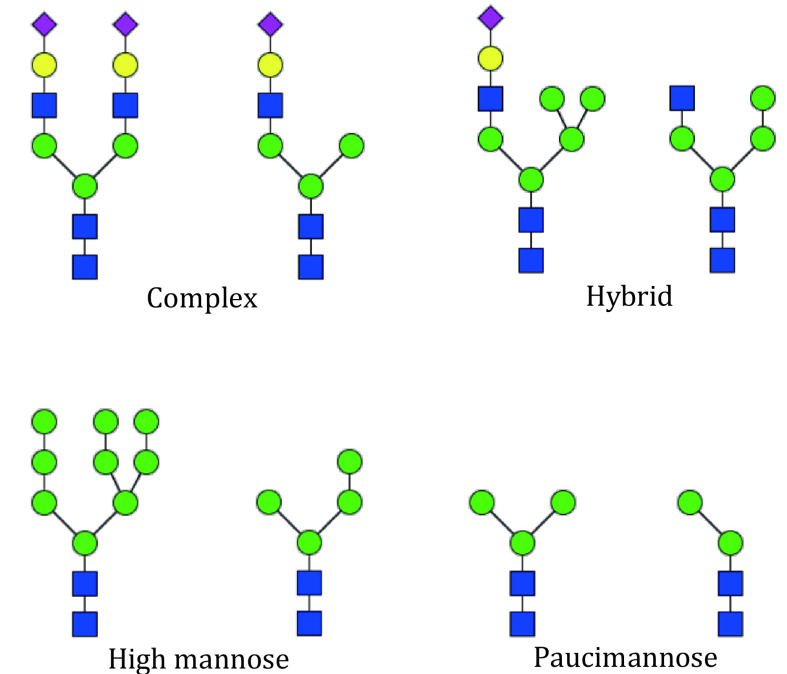
Classification of glycan types in StrucGP

Double click “MSFileName_result.xlsx”. In this table, the detailed information of each PSM is listed. A detailed explanation of each column can be found in the supplementary Table S1. It is worth noting that glycan types in StrucGP include high mannose, hybrid, complex, and paucimannose. Their differences are shown in Fig. 15.

### Result Visualization (time cost: minutes to hours)

#### Step 11: Open GlycoVisualTool

**Figure 16:**
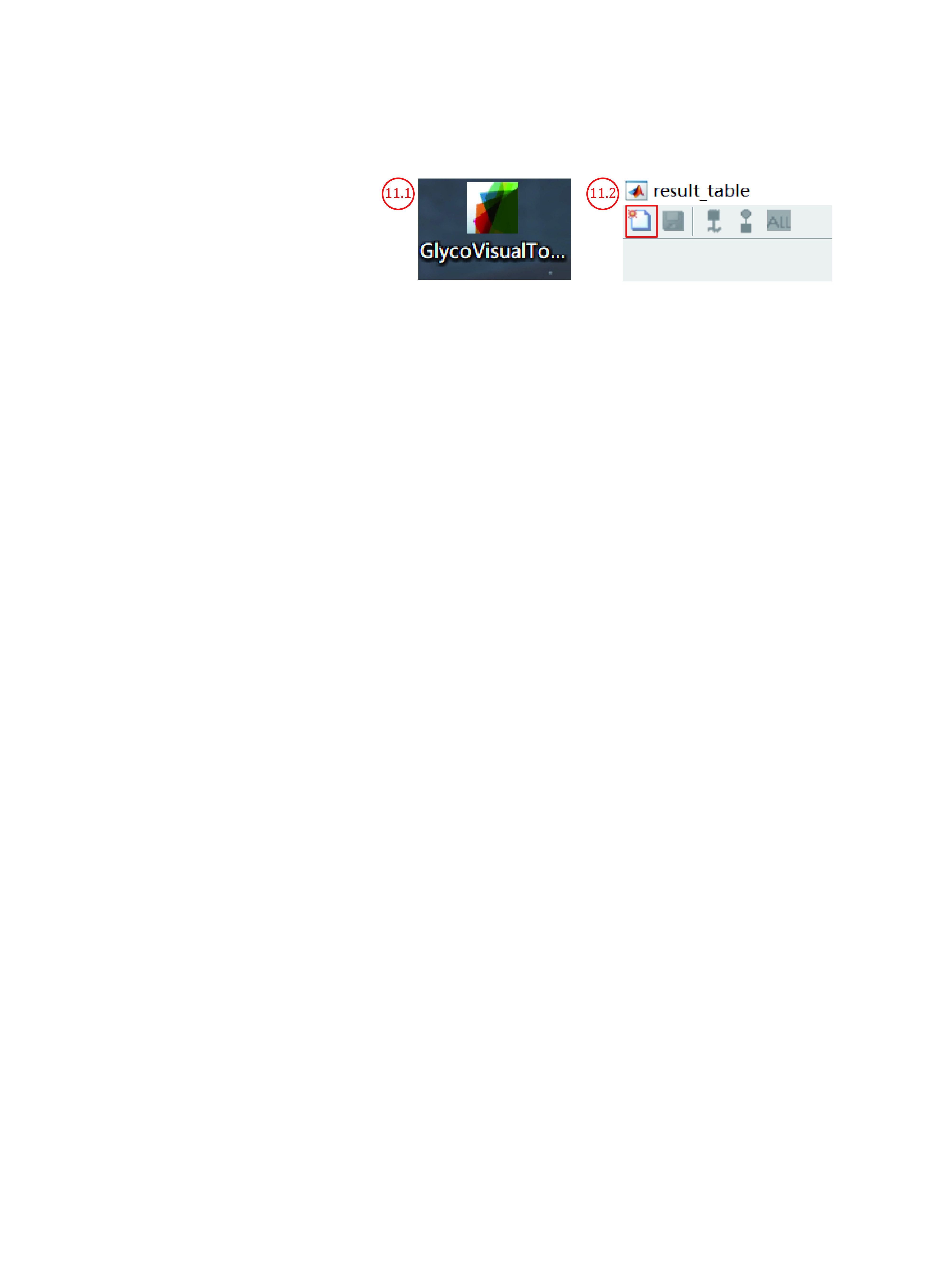
Open result file

11.1 Double click the icon of GlycoVisualTool (Fig. 16 left).

**[? TROUBLE SHOOTING]**

**Figure 17:**
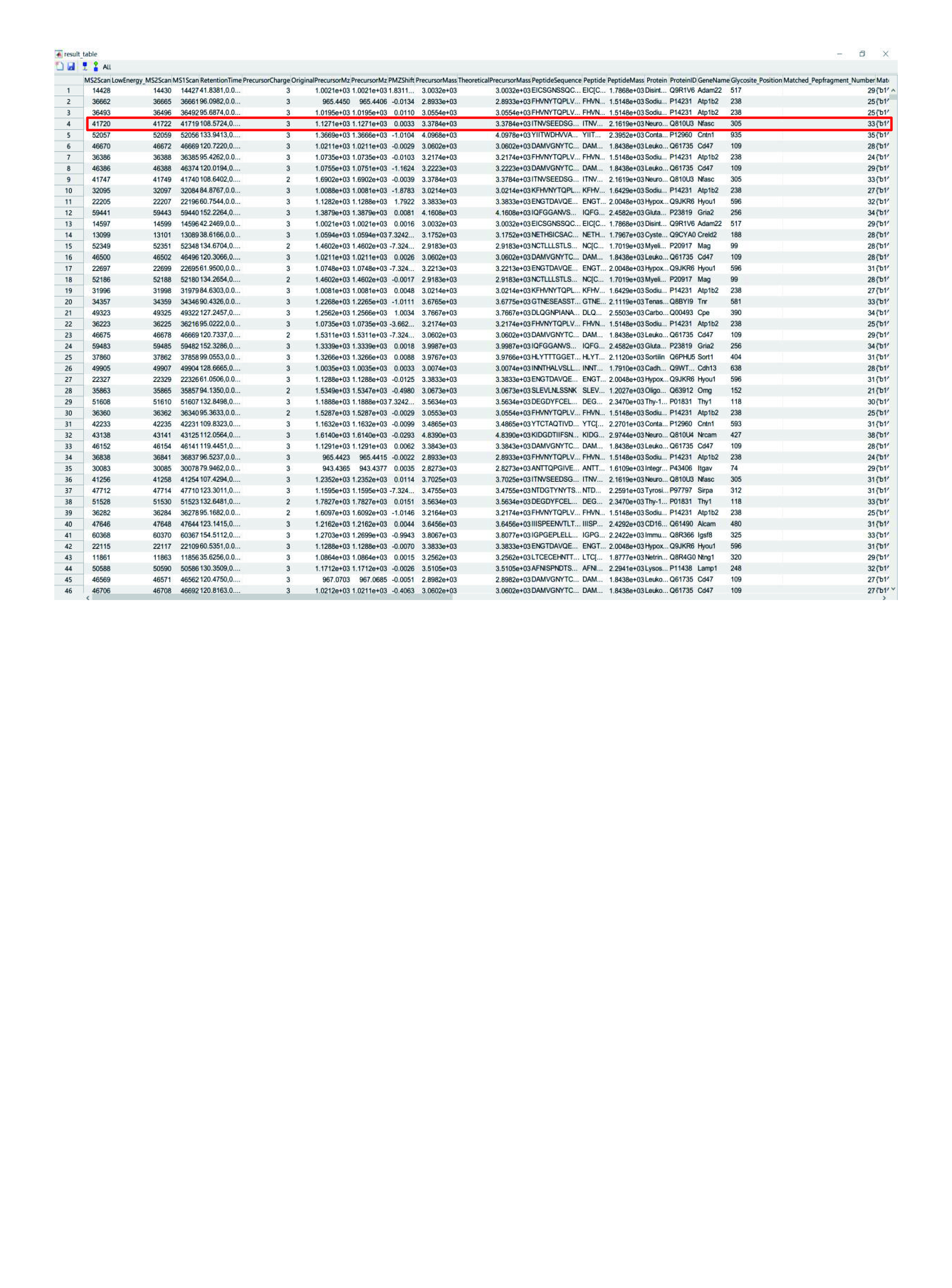
The interface of GlycoVisualTool after importing a .csv result

11.2 Click the first button to select the StrucGP result (.csv format) you want to visualize (Fig. 16 right), and you will see a result table as Fig. 17.

**Figure 18:**
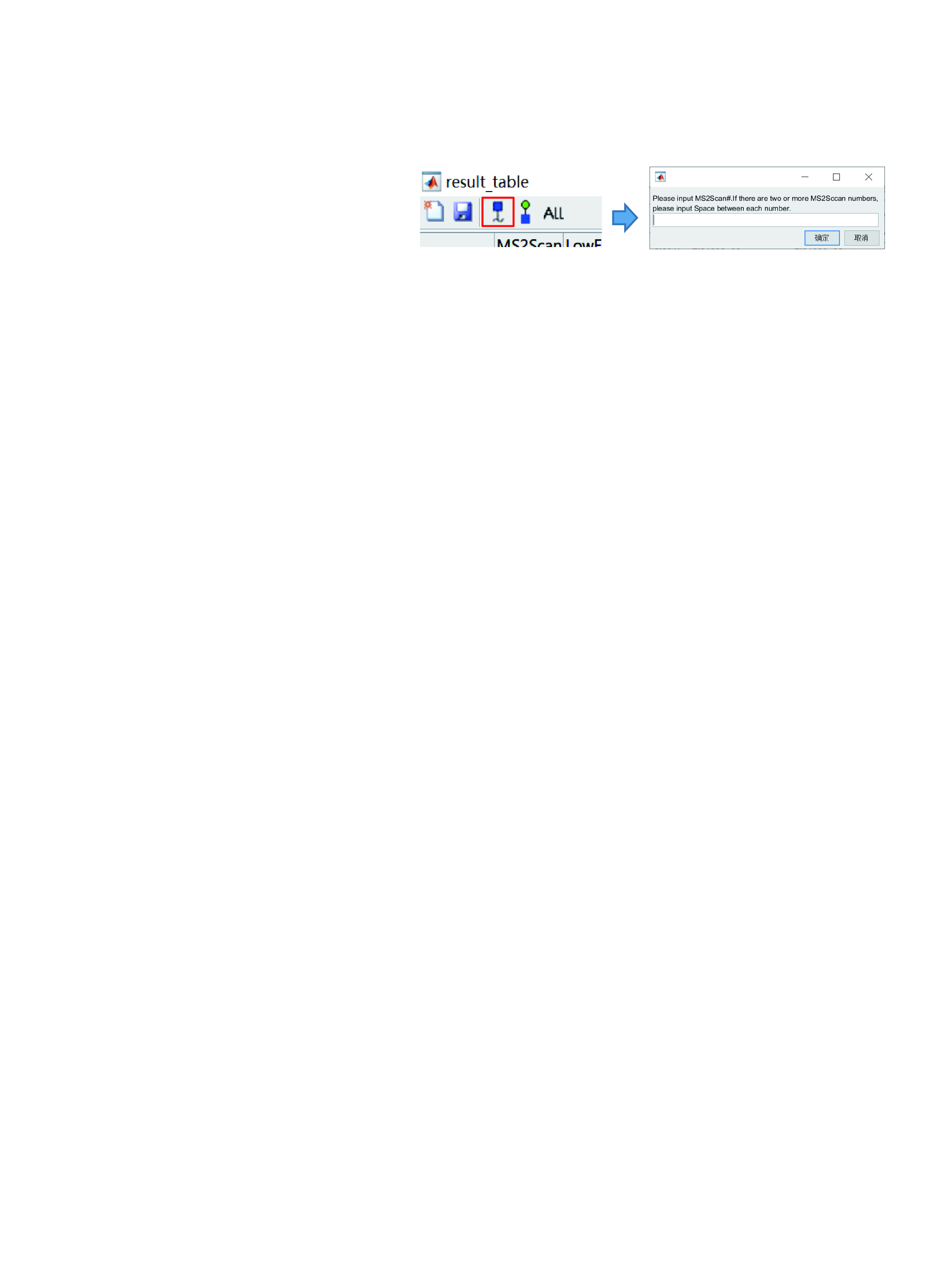
View spectrum of interest

11.3 To view annotated spectra of interest, click on the result table of GlycoVisualTool, then click on the row of the target spectrum (Fig. 17), or click the third button (Fig. 18 left) and specify the scan number in the pop-up window (Fig. 18 right).

**[NOTE]** It is important to note that “MS2Scan” means the scan number in the column “MS2Scan” (not “LowEnergy_MS2Scan”!) of the .xlsx format result. To open multiple spectra at once, enter spectra numbers and separate each of them with a space.

**Figure 19:**
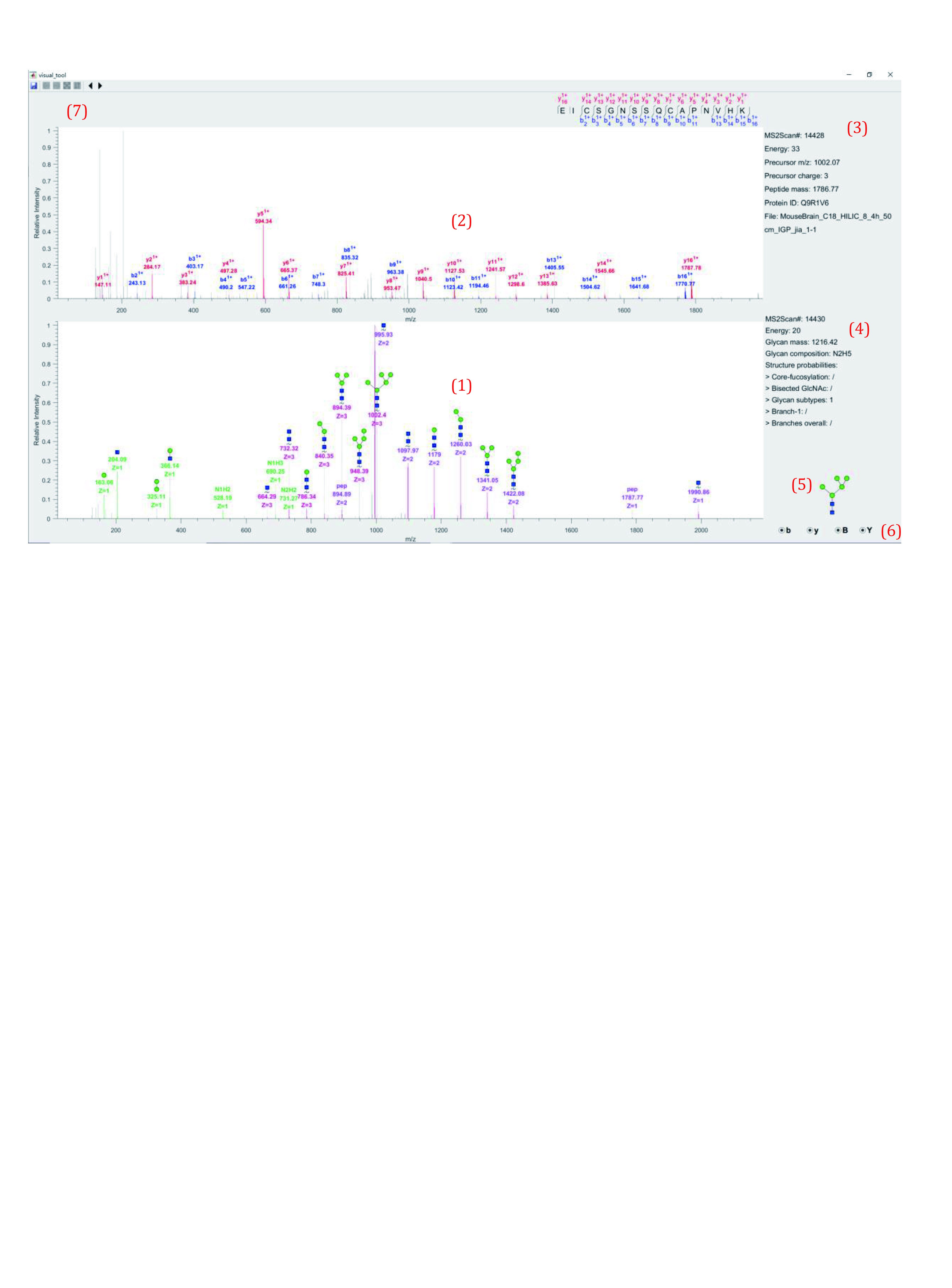
Spectra annotated by GlycoVisualTool

11.4 View annotated spectra (Fig. 19).

(1) Labeled B/Y ion peaks in the low energy spectrum. B ions are labeled with green lines and Y ions are labeled with purple lines. Besides, the *m/z* and charge of Y ions are also labeled with different colors to distinguish ions with different charges.

(2) Labeled b/y ion peaks in the high energy spectrum, b ions are labeled with blue lines and y ions are labeled with red lines. The *m/z* and charge are also labeled.

(3) Precursor and peptide information. Peptide mass and Protein ID are also provided.

(4) Glycan information. Glycan mass, composition, and structure probabilities.

(5) Glycan structure of current spectrum interpreted by StrucGP.

(6) The buttons to control the display/hide the labels of b/y and B/Y ions.

(7) The button to save this page as a figure.

**Figure 20:**
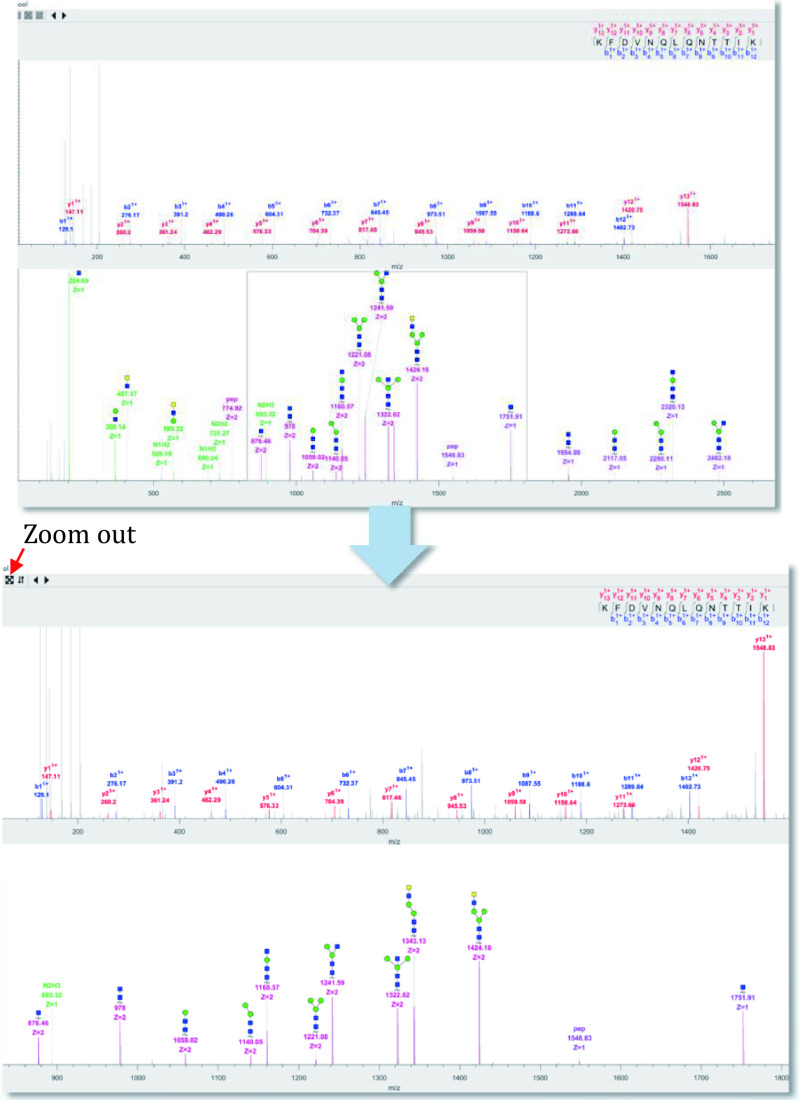
Zoom in on a specific area in the spectrum

**[Note]** You can drag the cursor through the peak area of interest to enlarge the selected area and click the fourth button to return to the original state (Fig. 20).

**[? TROUBLE SHOOTING]**

11.5 Check the quality of identified spectra.

**Figure 21:**
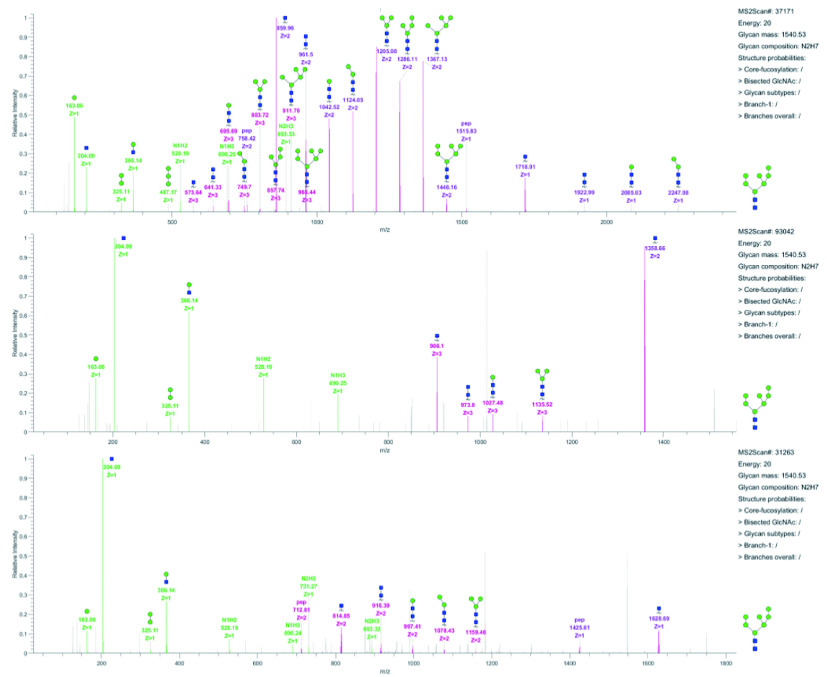
Three glycopeptide spectra with glycan HexNAc(2)Hex(7)

Generally, there are two main factors to judge whether an annotated glycan structure is reliable, *i*.*e*., the number and intensity of matched fragment ions and the “purity” of the glycan spectrum (the purity of each MS/MS spectra were evaluated based on the number of unmatched peaks in the spectrum. In the current version of the StrucGP, the more unmatched peaks exist in one spectrum, the less "purity" of the spectrum is). Figure 21 presents three glycopeptide spectra with the same glycan. The upper one has high credibility for its high intensity and complete B/Y ions; the middle spectrum lacks many Y ions and thus this identification is less reliable; the bottom spectrum is even less credible, except for the lack of Y ions, the intensity of matched Y ions is low and there are many unmatched peaks in the spectrum.

**Figure 22:**
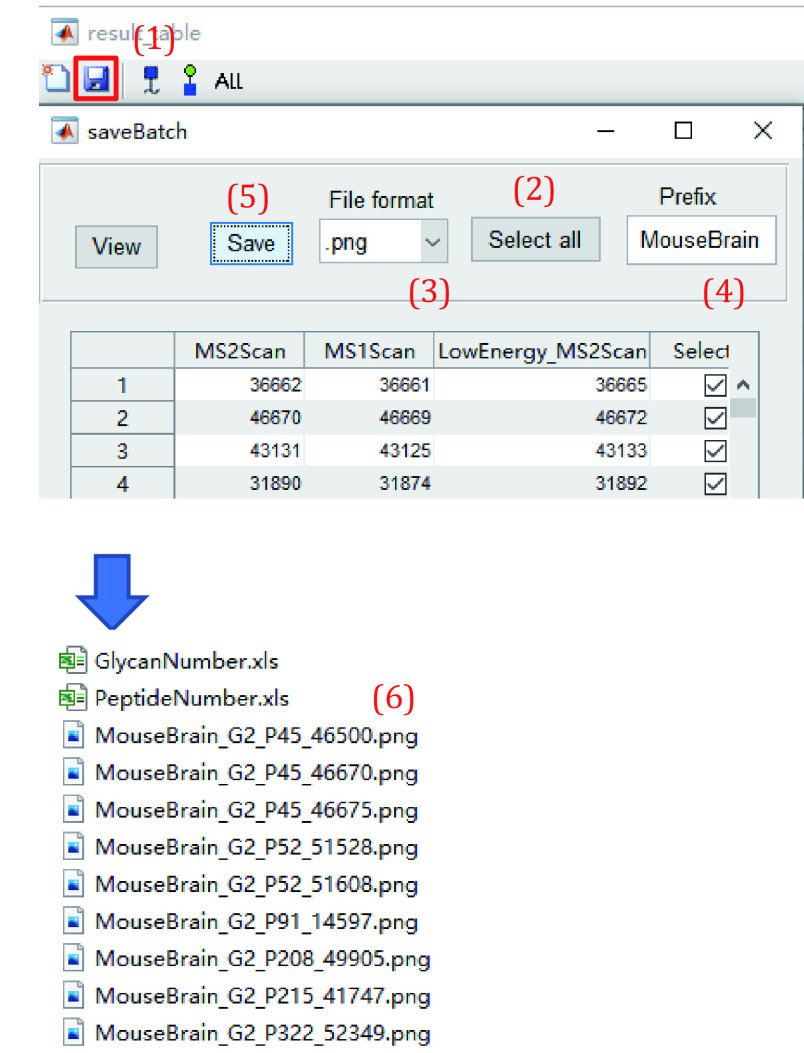
Save annotated spectra

11.6 Save all labeled spectra as figures (Fig. 22).

(1) Click the second button to save all labeled spectra and detailed information as figures.

(2) Click “Select all” to select all PSMs or click the box right to the desired scan number to select PSMs you want to save.

(3) Specify the format for saving spectrum.

(4) Enter characters as the prefix of the figures (*e*.*g*., MouseBrain).

(5) Click “Save”. It may take several hours to save thousands of spectra.

(6) The annotated spectra are named as “Profix_Gn_Pn_ScanNumber” where the “Gn” and “Pn” indicate the number of glycan structures and peptides in “GlycanNumber” and “PeptideNumber”, respectively.

**Figure 23:**
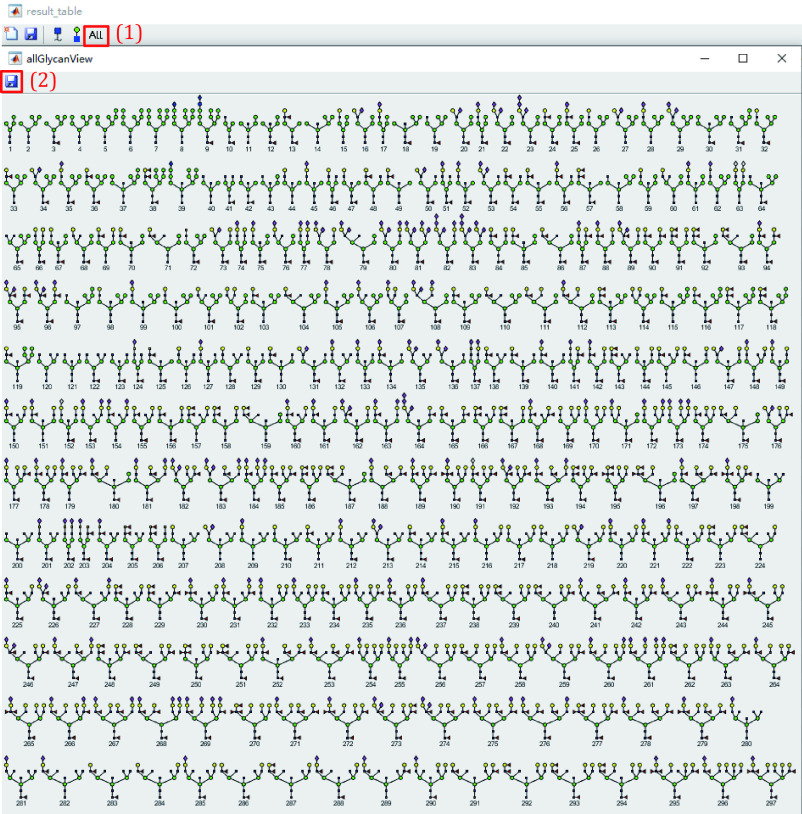
View and save all glycan structures in the current search result

11.7 View and save all identified glycan structures of the current result (Fig. 23).

(1) Click the fifth button to show all glycan structures of this result file.

(2) Click the top left button of the pop-up window to save these glycan structures.

**[Note]** The size of glycans is related to the number of all glycans, too many glycans in this table may cause an incomplete display.

**Figure 24:**
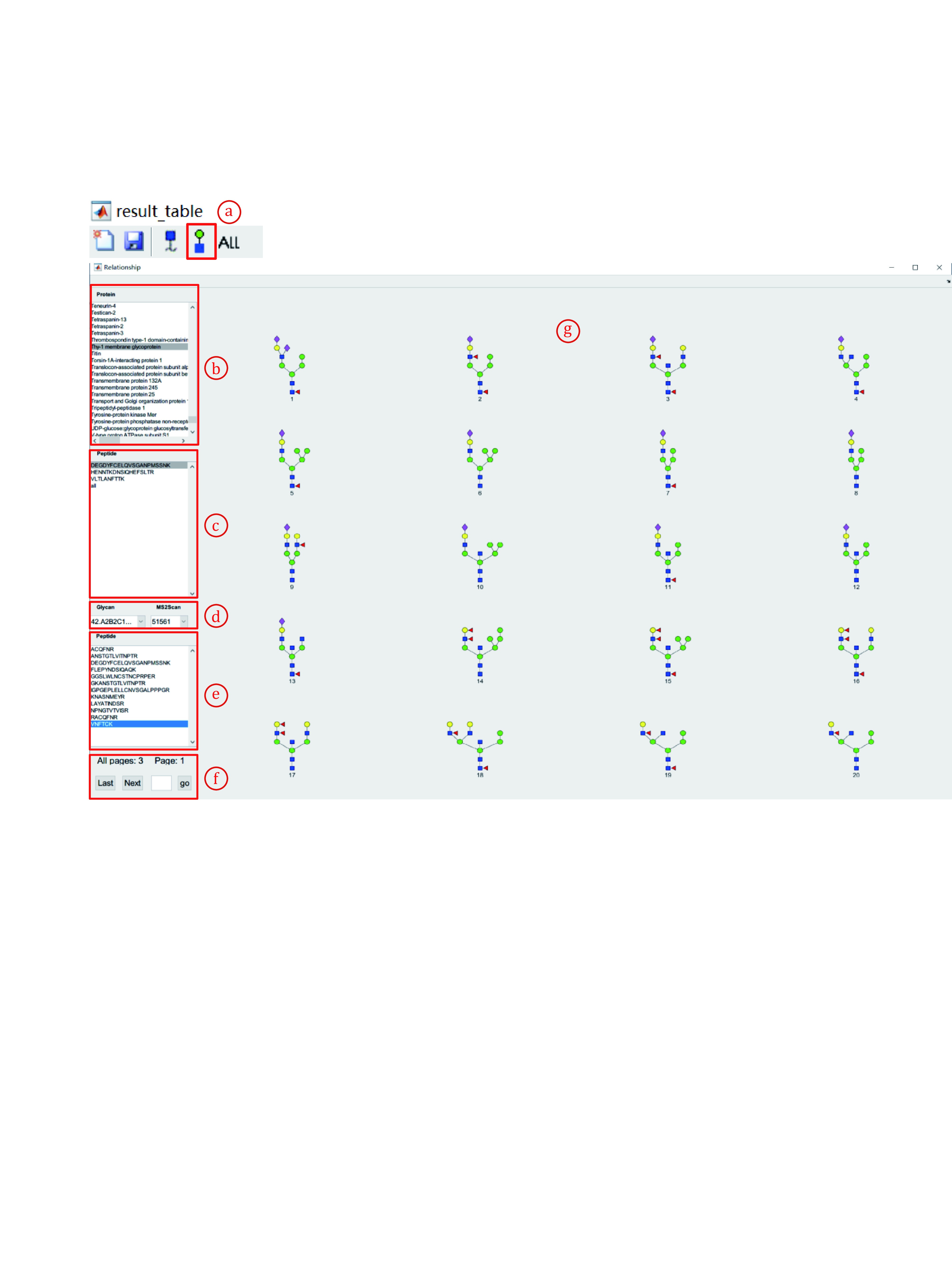
View glycans attached with a certain protein/peptide

11.8 Click the fourth button to view the relationship of all spectra in the result (Fig. 24, a). This function enables users to view all glycan structures for one specific peptide or view all peptides of a specified glycan structure (Fig. 24).

**[NOTE]** The startup process takes time (1–10 minutes). Please be patient.

(1) View all glycans attached to a selected protein/peptide. For example, to view all glycans attached on DGQLLPSSN^#^YSNIK from Neural cell adhesion molecule 1.

i. First, find Neural cell adhesion molecule 1 in the cell (b) and click it. The proteins are sorted alphabetically by the first letter of their name.

ii. Second, click “all” to view all glycans attached to Neural cell adhesion molecule 1. Select and click “DGQLLPSSN^#^YSNIK” to view glycans attached to this selected peptide (g).

iii. Glycans attached with “DGQLLPSSN^#^YSNIK” and their corresponding MS2Scan numbers are listed.

iv. All peptides modified with the selected glycans in **d** will be listed in the cell (e).

**Figure 25:**
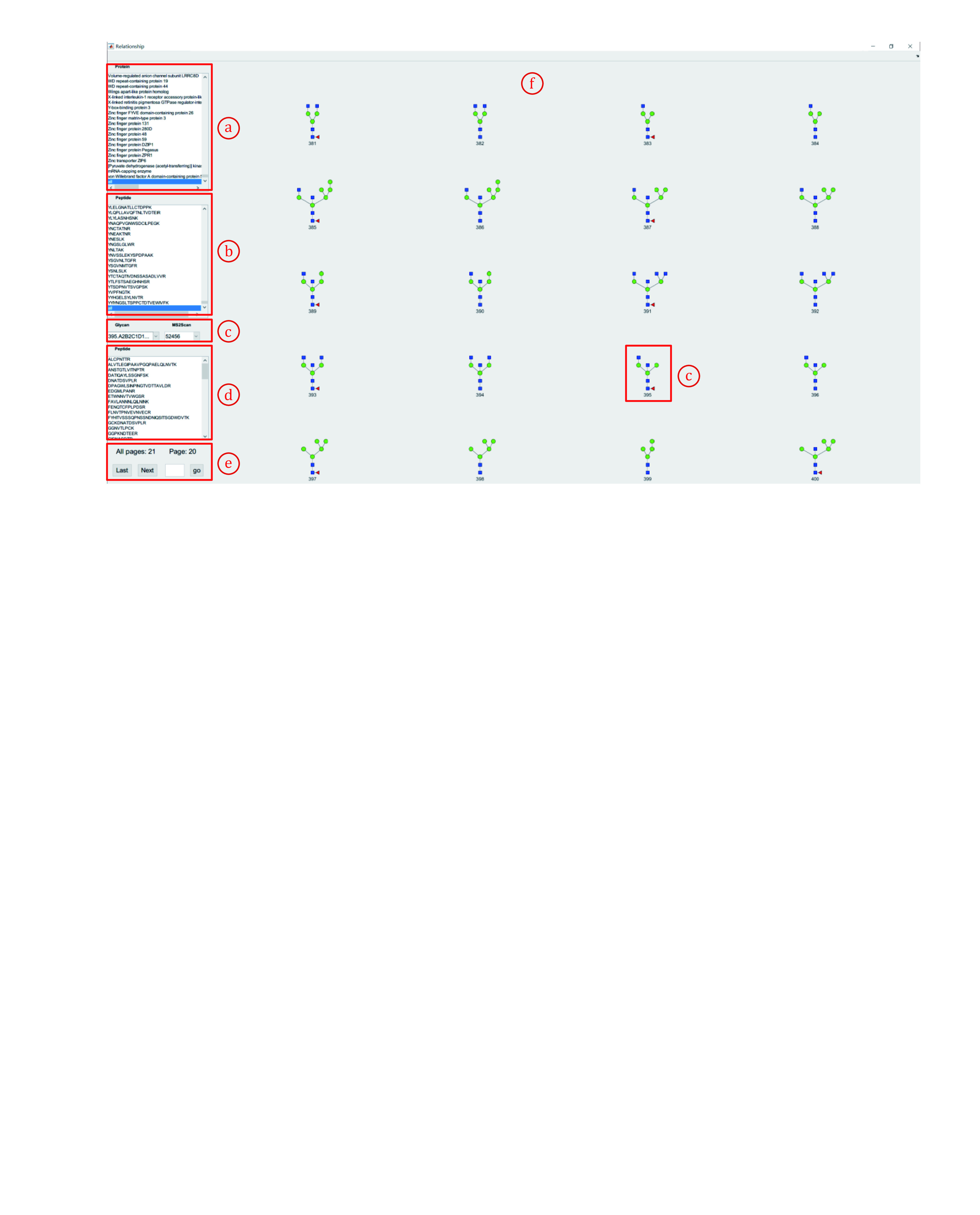
View all peptides modified with a certain glycan

(2) View all peptides with a certain glycan. For example, to view all peptides modified with “A2B2C1D1E2edD2dD1dcB5ba” (N4H3F1) (Fig. 25).

i. Select and click “all” in the cell (a).

ii. Select and click “all” in the cell (b).

iii. All glycans will be listed in the cell (c). All glycans will also be displayed as figures in the area (f).

iv. Switch the pages to find the No. of “A2B2C1D1E2edD2dD1dcB5ba”. You can find it on page 20 and its number is 395.

v. Find and click No.395 in the cell (c). All peptides modified with “A2B2C1D1E2edD2dD1dcB5ba” will be listed in the cell (d).

**[? TROUBLE SHOOTING]**

Troubleshooting advices can be found in Table 2.

**Table 2:** Troubleshooting

Step	Problem	Possible reason	Solution
Step 8	The console window shows “MemoryError” and process bar has been suspended for more than 10 minutes	Low computer memory (RAM) for running StrucGP	Set a lower “Threads” in Step 7.6, close programs that take up a lot of memory, and search again
Step 8	Process bar has been suspended for more than 10 minutes and the process shows done in the console window	Wrong high and low energy setting or inappropriate setting for screening glycopeptide spectra	Readjust parameters based on error messages and search again. If the error persists, contact us by taking screenshots of the console window and parameter settings and then sending them to sun_glycolab@126.com.
Step 11.1	Can’t open GlycoVisualTool	The version of MATLAB Runtime is not suitable	Reinstall GlycoVisualTool and MATLAB Runtime
Step 11.4	Incomplete display of glycan structures	The resolution of the screen does not match GlycoVisualTool	Adjust the resolution of the screen

## Conflict of interest

Jiechen Shen, Zexuan Chen and Shisheng Sun declare that they have no conflict of interest.
